# Review on Catalytic Oxidation of VOCs at Ambient Temperature

**DOI:** 10.3390/ijms232213739

**Published:** 2022-11-08

**Authors:** Rui Zhao, Han Wang, Dan Zhao, Rui Liu, Shejiang Liu, Jianfeng Fu, Yuxin Zhang, Hui Ding

**Affiliations:** 1 School of Environmental Science and Engineering, Tianjin University, Tianjin 300072, China; 2 College of Materials Science and Engineering, Chongqing University, Chongqing 400044, China

**Keywords:** nanocatalysts, single-atom catalysts, catalytic oxidation, volatile organic compound degradation, ambient temperature catalysis

## Abstract

As an important air pollutant, volatile organic compounds (VOCs) pose a serious threat to the ecological environment and human health. To achieve energy saving, carbon reduction, and safe and efficient degradation of VOCs, ambient temperature catalytic oxidation has become a hot topic for researchers. Firstly, this review systematically summarizes recent progress on the catalytic oxidation of VOCs with different types. Secondly, based on nanoparticle catalysts, cluster catalysts, and single-atom catalysts, we discuss the influence of structural regulation, such as adjustment of size and configuration, metal doping, defect engineering, and acid/base modification, on the structure–activity relationship in the process of catalytic oxidation at ambient temperature. Then, the effects of process conditions, such as initial concentration, space velocity, oxidation atmosphere, and humidity adjustment on catalytic activity, are summarized. It is further found that nanoparticle catalysts are most commonly used in ambient temperature catalytic oxidation. Additionally, ambient temperature catalytic oxidation is mainly applied in the removal of easily degradable pollutants, and focuses on ambient temperature catalytic ozonation. The activity, selectivity, and stability of catalysts need to be improved. Finally, according to the existing problems and limitations in the application of ambient temperature catalytic oxidation technology, new prospects and challenges are proposed.

## 1. Introduction

Volatile organic compounds (VOCs) are important precursors for ozone generation, photochemical reactions, and secondary organic aerosols that cause serious deterioration of the atmospheric environment [1]. VOCs come from a wide range of natural sources and anthropogenic sources. Natural sources mainly include vegetation emission. Anthropogenic sources are more complex, including but not limited to transportation, chemical production, food processing, paint drying, and petroleum refining, which have the characteristics of high emission intensity, wide sources, multiple types, and large fluctuations [2]. VOCs working directly as toxic substances cause serious environmental pollution, also causing carcinogenesis, teratogenesis, or mutagenesis to the human body [3]. At present, the health of the atmospheric environment has attracted extensive attention, and optimizing VOC treatment technology has also become an environmental problem that researchers need to solve urgently.

The existing VOC treatment methods can be divided into recovery technologies (adsorption, absorption, membrane separation, condensation, etc.) and destruction technologies (catalytic oxidation, thermal incineration, biodegradation, photocatalytic degradation, low-temperature plasma technology, etc.) [4,5,6,7,8]. Since various pollutants have different elimination difficulties, concentrations, and sources, each technology has corresponding advantages and limitations [9]. Recovery technology is suitable for the removal of low-concentration VOCs, which has advantages of recycling, high efficiency, and economy. However, it needs further desorption treatment, and has the possibility of secondary pollution. By contrast, destruction technology applies to VOCs with a wide concentration range, which can completely decompose VOCs into harmless small-molecular substances. However, the disadvantages of destruction technology are also obvious, such as high energy consumption and certain safety risks [10,11]. Under the strict standards of VOC emission, condensation, and adsorption, it is difficult for direct combustion and other traditional methods to achieve an ideal treatment effect. Combining the economic and technical advantages of various treatments, catalytic oxidation is considered one of the most effective methods to eliminate VOCs due to its low cost and high removal rate [12]. Furthermore, ambient temperature catalytic oxidation technology (one atmospheric pressure, ambient temperature) as a more environmentally friendly and clean treatment technology breaks through the inertial thinking that VOC catalytic oxidation requires energy input, and can achieve effective degradation of VOCs under normal conditions without high temperature, high pressure, electro-discharge, and ultraviolet light.

To meet such demand, it is important to identify suitable catalysts to realize ambient temperature catalytic oxidation. Some articles have addressed the developments in catalytic technology toward the removal of VOCs [10,11,13,14,15,16,17,18,19,20]. Nevertheless, these works had limited scope as they only focused on low-temperature catalytic processes based on specific types of catalysts (e.g., Mn-based oxide catalysts) or pollutant molecules (e.g., formaldehyde) [14,15,16]. Although there have been some articles that summarize the research and development of low-temperature catalytic VOCs in recent years, the activity of general catalysts still cannot meet the requirements of ambient temperature oxidation [19,20]. Ozone-assisted, photo-assisted, and thermal-assisted methods have been used to enhance catalytic activity [11,18]. Therefore, researchers have analyzed the structure–activity relationship of catalysts, the electronic structure, and the coordination environment of active sites [10,17]. In addition, to the best of our knowledge, no attempt has been made in the literature to offer a comprehensive review on the application of catalytic oxidation at ambient temperature to specifically describe the modification of ambient temperature catalysts.

This review focuses on the progress of catalytic oxidation of VOCs at ambient temperature in recent decades. Firstly, we summarized the catalytic oxidation effects of common volatile organic compounds, including aromatic hydrocarbon, aliphatic hydrocarbon, oxygen-containing VOCs, and chlorine/sulfur-containing VOCs, at ambient temperature. Secondly, this review briefly describes the generation mechanism of reactive oxygen species with important oxidative activity in catalyzed reactions. In order to reduce the temperature of VOC catalytic oxidation, researchers are committed to improving the catalytic effect by adjusting the catalyst structure and optimizing the catalytic reaction conditions. Therefore, according to the size level of active components, the catalysts are divided into nanoparticle catalysts, cluster catalysts, and monatomic catalysts. We further discuss the influence of structural regulation such as catalyst size and configuration, metal doping, defect engineering, acid/base modification, and process optimization conditions such as ozone assistance and humidity adjustment. Finally, we present some challenges of ambient temperature catalytic oxidation technology and look forward to future development in this field.

## 2. Catalyst Sorts and Catalytic Elimination of VOCs

According to the definition by the World Health Organization (WHO), VOCs refer to a group of organic compounds with boiling points in the range of 50~260 °C under atmospheric pressure (101.325 KPa) [21]. For the molecular structure, VOCs can be divided into aromatic hydrocarbon, aliphatic hydrocarbon, oxygen-containing VOCs, and chlorine/sulfur-containing VOCs.

### 2.1. Aromatic Hydrocarbon Elimination

Aromatic hydrocarbons are typical representative substances of VOCs emitted by industry, among which benzene, toluene, ethylbenzene, and xylene are the main pollutants [22]. Aromatic VOCs have a wide range of emission sources, strong toxicity, and pose significant harm to the environment and the human body [23]. As a collection of benzene series (BTEX), aromatic VOCs have benzene rings with stable structure, low energy, and are hardly degradable. Therefore, many researchers are committed to building high-performance catalysts to realize catalytic oxidation at room temperature. As shown in Table 1, existing studies focus on the catalytic oxidation of benzene and toluene at ambient temperature. Most of the catalysts are based on metal or metal oxides as active sites to improve the activation ability of reactant molecules. A variety of silicon-based materials, activated carbon, and metal oxides as catalyst support provide a large enough surface area and molecular adsorption energy.

### 2.2. Aliphatic Hydrocarbon Elimination

Aliphatic hydrocarbon is an important component of petroleum and natural gas, widely used as engine fuel and chemical raw materials [18,24]. Due to incomplete combustion and disorganized emission, aliphatic hydrocarbons often appear in automobile and industrial exhaust, resulting in serious photochemical smog, crop yield loss, and chronic respiratory diseases [25,26]. Aliphatic VOCs can be divided into alkanes, alkenes, and alkynes according to the type of bond between carbon atoms. The high-energy C–H bond in short-chain alkanes endows them with stable chemical properties, so they are difficult to oxidize [27,28,29]. Therefore, the application of catalytic oxidation of aliphatic hydrocarbons at room temperature is less common. As shown in Table 1, studies focus on the degradation of unsaturated aliphatic hydrocarbons by noble metal catalysts. In the degradation of aliphatic VOCs, increasing the activities of C-H, C-C, C=C, and C≡C and promoting oxidative cleavage are the key issues to achieving catalytic oxidation at room temperature.

### 2.3. Oxygen-Containing Elimination

The formation process of oxygen-containing volatile organic compounds (OVOCs) is complex; one part is emitted from anthropogenic or plant sources, and the other part is completed by secondary conversion of anthropogenic sources or biological sources in the atmosphere [30,31]. OVOCs are mainly composed of aldehydes, ketones, alcohols, ethers, small molecular organic acids, and organic esters. As shown in Table 1, so far, many reports can realize the catalytic oxidation of OVOCs at room temperature. The catalysts used include noble metal nanoparticle catalysts, noble metal single-atom catalysts, transition metal oxide catalysts, and metal composite nanosheet catalysts.

### 2.4. Chlorine/Sulfur-Containing Elimination

Chlorine-containing volatile organic compounds (CVOCs) are widely used in chemical production, and enter the atmosphere through volatilization, leakage, and industrial waste gas emission during production [32]. Sulfur-containing volatile organic compounds (SVOCs), which occur in major petroleum products and industrial waste gases, are a kind of highly active volatile organic compounds with an unpleasant smell and can cause serious harm by irritating the respiratory tract and skin, leading to human poisoning [33]. The electronegativity of the Cl element in CVOCs is very strong, which makes it difficult for C-Cl to break without high-temperature assistance. However, the removal of CVOCs by high-temperature incineration is prone to produce highly toxic polychlorinated by-products (including dioxins) [34]. In addition, chloride ions tend to react with the active site of the catalyst, resulting in catalyst deactivation and poisoning [35]. In the process of catalytic decomposition of SVOCs, the catalyst also suffers from carbon or sulfur poisoning [36,37]. Thus, the development of normal temperature catalysts with high activity and high toxicity resistance is a research hotspot in the catalytic oxidation of CVOCs and SVOCs.

**Table 1:** Summary of typical catalysts for catalytic elimination of VOCs at ambient temperature.

Catalyst		Reaction Mixture	Reaction Conditions	Reaction Temperature	Conversion	CO_2_ Selectivity	Ref.
**Aromatics**							
Benzene	MnO_2_/ZSM-5	C_6_H_6_: 30 ppm O_3_: 450 ppm	GHSV ^1^: 48,000 h^−1^ RH ^3^: 50%	25 °C	100%	-	[38]
	MnO_x_/AC	C_6_H_6_: 30 ppm O_3_: 300 ppm	GHSV: 28,000 h^−1^ RH: 50%	25 ± 1 °C	100%	61.9%	[39]
Toluene	Pt/MnO_x_-T	C_7_H_8_: 30 ppm O_3_: 300 ppm	WHSV ^2^: 60 L g^−1^ h^−1^	RT ^4^	98%	90%	[40]
	Y/La-MnO_2_	C_7_H_8_: 10 ppm	WHSV: 60 L g^−1^ h^−1^	40 °C	100%	100%	[4]
	Pt/TiO_2_	C_7_H_8_: 250 mg m^−3^ O_3_: 2600 mg m^−3^	GHSV: 30,000 h^−1^	RT	65%	100%	[41]
	Mn/Al_2_O_3_	C_7_H_8_: 50 ppm O_3_: 1000 ppm	WHSV: 120 L g^−1^ h^−1^	RT	98.94%	~47%	[42]
	MnO_x_/Al_2_O_3_	C_7_H_8_: 120 ppm O_3_: 1000 ppm	WHSV: 300 L g^−1^ h^−1^	25 °C	80%	-	[43]
	ZnFe_2_O_4_/ɣ-Al_2_O_3_	C_7_H_8_: 49 ppm O_3_: 97 ppm	GHSV: 1500 h^−1^	20 °C	~100%	100%	[44]
	MnO_x_/MCM-41	C_7_H_8_: 100 ppm O_3_: 1000 ppm	-	20 °C	100%	73%	[45]
	Pt-Ce/BEA	C_7_H_8_: 22 ppm O_3_: 300 ppm	WHSV: 60 L g^−1^ h^−1^	30 °C	95%	56%	[46]
	3D-NiO_1_-δ/NF	C_7_H_8_: 100 ppm O_3_: 350 ppm	GHSV: 10,000 h^−1^ RH: 50%	25 °C	100%	70.6%	[47]
	Mn/AC-0.43FN	C_7_H_8_: 100 ppm O_3_: 2100 ppm	WHSV: 300 L g^−1^ h^−1^	30 °C	~87%	36%	[48]
	Mn(OH)F/Ni	C_7_H_8_: 100 ppm O_3_: 1000 ppm	GHSV: 6000 h^−1^ RH: 50%	25 °C	100%	-	[49]
**Aliphatic hydrocarbons**							
Ethylene	Ag/ZSM-5	C_2_H_2_: 100 ppm	WHSV: 7500 mL g^−1^ h^−1^	RT	100%	100%	[50]
	5%Ag/ZSM-5	C_2_H_2_: 100 ppm	WHSV: 7500 mL g^−1^ h^−1^	RT	100%	100%	[51]
	Pt/F-ZSM-5	C_2_H_2_: 100 ppm	WHSV: 7500 mL g^−1^ h^−1^	25 °C	100%	100%	[52]
**OVOCs**							
Methanol	Pt/FeO_x_	CH_4_O: 380 ppm O_3_: 200 ppm	GHSV: 24,000 h^−1^ RH: 30%	30 °C	100%	100%	[53]
Isopropanol	Na-Pt/TiO_2_	C_3_H_8_O: 75.00 ppm O_3_: 105.30 ppm	WHSV: 16,920 mL g^−1^ h^−1^	35 °C	100%	91.34%	[54]
Oxalic acid	Mn-C@Fe	H_2_C_2_O_4_: 40 mg L^−1^ O_3_: 0.36 mM	WHSV: 120 L g^−1^ h^−1^	25 ± 0.5 °C	90.9%	-	[55]
Ethyl acetate	Pd/ACF-fiber	C_4_H_8_O_2_: 350 mg/m^3^ O_3_: 1000 mg/m^3^	GHSV: 30,000 h^−1^	30~50 °C	60%	92%	[56]
	β-MnO_2_	C_4_H_8_O_2_: 500 ppm O_3_: 1000 ppm	GHSV: 10,000 h^−1^	RT	100	70%	[57]
Formaldehyde	Pd/TiO_2_	HCHO: 140 ppm	GHSV: 95,000 h^−1^	RT	100%	-	[58]
	α-MnO_2_	HCHO: 60 ppm O_3_: 230 ppm	WHSV: 120 L g^−1^ h^−1^ RH: 50%	RT	90%	100%	[59]
	3D-MnO_2_	HCHO: 100 ppm	WHSV: 180 L g^−1^ h^−1^ RH: 65%	RT	45%	100%	[60]
	MnO_x_	HCHO: 80 ppm	GHSV: 180,000 h^−1^ RH 33%	RT	93%	-	[61]
	MnO@C	HCHO: 60 ppm O_3_: 180 ppm	WHSV: 60 L g^−1^ h^−1^ RH: 50%	30 °C	100%	100%	[62]
	MnCeO_x_	HCHO: 55 ppm O_3_: 165 ppm	WHSV: 210,000 h^−1^ RH: 60%	25 °C	100	70%	[63]
	3D-NiCo_2_O_4_	HCHO: 200 ppm	GHSV: 60,000 h^−1^	25 °C	95.30%	100%	[64]
	MnO_x_/CVC-TiO_2_	HCHO: 45 ppm O_3_: 225 ppm	GHSV: 20,000 h^−1^ RH: 50~80%	25 °C	100%	100%	[65]
	Au/Co-MgAl LDH	HCHO: 80 ppm	WHSV: 18 L g^−1^ h^−1^	RT	96.2%	100.00%	[66]
	Pt/NiAl-LDHs	HCHO: 223 ppm	-	RT	92.40%	100%	[67]
	Pt/SnO_x_	HCHO: 172 ppm	-	RT	87%	100%	[68]
	K-Pt/NaY	HCHO: 300 ppm	RH: 35~51%	RT	98%	100%	[69]
	Pt/NiO	HCHO: 200 ppm	-	RT	89%	-	[70]
	NiO	HCHO: 60 ppm O_3_: 180 ppm	WHSV: 60 L g^−1^ h^−1^ RH: 50%	30 °C	100%	100%	[71]
	Au-Ce_3_Co/GA	HCHO: 50 ppm	WHSV: 20 L g^−1^ h^−1^	60 °C	100%	100%	[72]
	Pt/Co_3_O_4_	HCHO: 210 ppm	-	RT	91.40%	100%	[73]
	Pt-Ni/ZSM-5	HCHO: 50 ppm	WHSV: 30 L g^−1^ h^−1^ RH: 35%	30 °C	90%	100%	[74]
	Pt/Al_2_O_3_	HCHO: 160 ppm	-	RT	62.50%	100%	[75]
	Pt/NiO	HCHO: 200 ppm	RH: 50%	RT	90%	100%	[76]
Acetone	MnO_x_/Al_2_O_3_	C_3_H_6_O: 120 ppm O_3_: 1150 ppm	WHSV: 600 L g^−1^ h^−1^	RT	95%	-	[77]
	Mn-Co/γ-Al_2_O_3_	C_3_H_6_O: 150 ppm O_3_: 1200 ppm	WHSV: 231 L g^−1^ h^−1^	RT	84%	-	[78]
	CoO/γ-Al_2_O_3_	C_3_H_6_O: 150 ppm O_3_: 1200 ppm	WHSV: 230 L g^−1^ h^−1^	RT	85%	-	[79]
**Cl, S-containing VOCs**							
Chlorobenzene	MnO_x_/CNT	C_6_H_5_Cl: 50 ppm O_3_: 2300 ppm	WHSV: 24 L g^−1^ h^−1^	80 °C	95%	100%	[80]
Methyl mercaptan	α-MnO_2_	CH_3_SH: 70 ppm O_3_: ~933 ppm	WHSV: 120 L g^−1^ h^−1^	RT	100%	-	[81]
	Ag/MnO_2_	CH_3_SH: 70 ppm O_3_: 700 ppm	WHSV: 60 L g^−1^ h^−1^	RT	95%	-	[82]
	CuO/Vo-MnO_2_	CH_3_SH: 80 ppm O_3_: 200 ppm	GHSV: 60,000 h^−1^	RT	99%	-	[83]

^1^ GHSV: gas hourly space velocity; ^2^ WHSV: weight hourly space velocity; ^3^ RH: relative humidity; ^4^ RT: room temperature.

## 3. Reaction Mechanism for VOC Catalytic Oxidation

The kinetic mechanisms proposed for the catalytic oxidation of VOCs are generally divided into Langmuir Hinshelwood (L-H), Eley Rideal (E-R), and Mars van Krevelen (MVK). The L-H mechanism considers that the reaction occurs between adsorbed oxygen species and adsorbed VOCs. This model can be further subdivided into L-H single-site (LHs) and L-H dual-site (LHd) based on the adsorption competition of reactants at active sites [84,85]. The E-R mechanism and L-H mechanism are both surface reaction models, but the mechanism of E-R is different in the reaction process that occurs between adsorbed oxygen species and gaseous reactant molecules [18]. Additionally, the MVK mechanism, as the most commonly used catalytic oxidation model, assumes that the reaction proceeds between the oxygen-rich sites and adsorbed VOCs. The key to this mechanism is the reoxidation of catalysts [20]. The oxidation mechanism of L-H and E-R depend on the adsorbed oxygen species whose source is oxygen or ozone in the reaction environment on the catalyst surface. Nevertheless, the MVK mechanism relies on chemically chemisorbed oxygen or lattice oxygen in the catalyst, rather than oxygen in the gas phase [19,77].

Clarifying the oxidation mechanism of VOCs in the catalytic process is a fundamentally effective way to realize the efficient and stable degradation of pollutants at room temperature. Unfortunately, due to different properties of pollutants and different surface reaction conditions of catalysts, it is difficult to define a general mechanism for the complete oxidation of VOCs. Therefore, even though researchers have explored the catalytic oxidation mechanism of VOCs, the above three models still cannot completely generalize the catalytic oxidation at ambient temperature. In essence, the catalytic degradation of VOCs is an oxidation reaction. It is not difficult to find that the oxidation activity of the ambient temperature catalytic reaction mainly comes from the reactive oxygen species. Accordingly, the formation mechanism of reactive oxygen species is important. The common species include superoxide radical (·O_2_^−^), hydroxyl radical (·OH), and singlet oxygen (^1^O_2_), which are mainly formed by the activation of ozone, oxygen and water in metal active sites, defects and acid/base sites.

Under the action of metal active sites (M_as_), O_2_ or O_3_ molecules are dissociated into active oxygen atoms, which further interact with the adsorbed H_2_O molecules to form reactive oxygen species, as shown in Equations (1)–(3):

M_as_ + O_2_ → 2 O^−^ (1)

M_as_ + O_3_ → O^−^ + O_2_ (2)

O^−^ + H_2_O → 2 ·OH (3)

**Figure 1:**
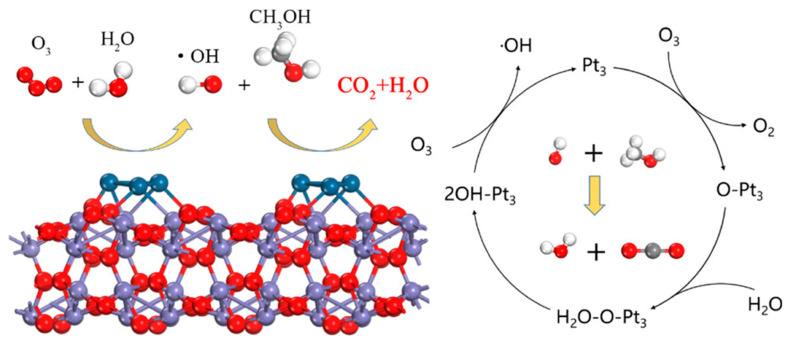
Reaction mechanism of Pt/FeO_x_ catalyst for methanol catalytic ozonation at ambient temperature. Reprinted with permission: Copyright American Chemical Society 2020 [53].

Yan et al. [67] prepared Pt/NiAl LDHs catalyst. Pt nanoparticles on its surface acted as active sites to activate O_2_ molecules and reacted with H_2_O molecules to form hydroxyl groups, which could first oxidize formaldehyde molecules into formate species, and then further fully oxidized them into CO_2_ and H_2_O. Furthermore, Tian et al. [53] discussed different adsorption morphologies of O_3_ molecules through theoretical calculation and found that O_3_ molecules could form O_bri_ and O_top_, two kinds of surface-adsorbed oxygen atoms on Pt_3_ clusters. Comparing the adsorption energy and energy barrier on different adsorbed oxygen structures, it could be determined that the adsorption energy of H_2_O molecules in the O_top_ is low, which led to the stable adsorption structure and was conducive to the formation of surface hydroxyl radicals by crossing the extremely low energy barrier (Figure 1).

Under the action of oxygen vacancy (V_O_), the mechanism of O_2_ producing free radicals is consistent with that of M_as_. O_2_ is adsorbed and dissociated by V_O_ to form abundant reactive oxygen species O^−^, which can combine with H_2_O molecules to produce ·OH [86]. However, the mechanism of radicals produced with O_3_ is different. One atom of O_3_ binds to V_O_, and V_O_ transfers two electrons to the O atom to form adsorbed oxygen O^2−^, releasing an O_2_. Further new O_3_ molecules combine with O^2−^ to form peroxide species O_2_^2−^, which can decompose into an oxygen molecule and restore the oxygen vacancy [87], as shown in Equations (4)–(6):

V_O_ + O_3_ → O^2−^ + O_2_ (4)

O_3_ + O^2−^ → O_2_^2−^ + O_2_ (5)

O_2_^2−^ → O_2_ + V_O_ (6)

**Figure 2:**
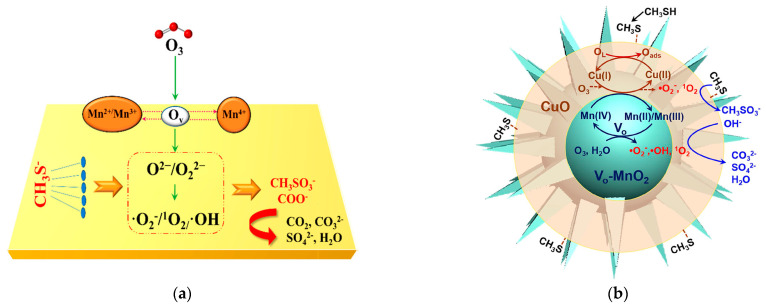
Reaction mechanism of methyl mercaptan catalytic ozonation at ambient temperature: (**a**) use of α-MnO_2_ catalyst. Reprinted with permission: American Chemical Society 2020 [81]; (**b**) use of CuO/V_O_-MnO_2_ catalyst, reprinted with permission: Elsevier 2020 [83].

In detail, He et al. [81] explored the mechanism of reactive oxygen species in oxygen vacancies (Figure 2a). The MnO_2_ catalyst with abundant oxygen vacancies promoted the adsorption of O_3_ and activated it into intermediate peroxide species (O^2−^/O_2_^2−^), which then interacted with H_2_O to conduct charge transfer, and finally formed reactive oxygen species (·OH, ·O_2_^−^, ^1^O_2_), so as to achieve efficient degradation of methyl mercaptan at room temperature. In addition, Yang et al. [83] explored the reaction mechanism of methyl mercaptan degradation by radicals using defect engineering in CuO-doped MnO_2_ (Figure 2b). Under the wet condition, surface hydroxyl groups (-OH) were spontaneously generated on oxygen vacancies and Mn-O bonds. Single-bonded hydroxyl groups can trap O_3_ to form -OH_2_ and O_2_^−^, accompanied by the formation of O_2_. The subsequent release of ·O_2_^−^ acted as the reactive oxygen species to oxidize methyl mercaptan. Finally, -OH_2_ on the catalyst surface continued to capture O_3_ and release HO_3_·. As an unstable radical, HO_3_· was further decomposed into ·OH and O_2_. The whole oxidation process was cyclic and sustainable. When ·OH contacted with ·O_2_^−^, ^1^O_2_ can be produced, which acted together with ·OH and ·O_2_^−^ to oxidize methyl mercaptan.

According to existing studies, the acidity of catalysts significantly affects the degradation performance of VOCs, which can promote the decomposition of O_3_ and generate reactive oxygen species [88,89]. It is generally believed that the mechanism of radical production at acid sites is consistent with the role of metal active sites. Using the Mg(OH)F/Ni catalyst, Zhu et al. [49] found that Lewis and Brønsted acid sites jointly catalyzed O_3_ to generate higher activity ·OH. There were two free radical production paths:

Path 1:

Mg(OH)F + O_3_ → O_3_···Mg(OH)F (7)

O_3_···Mg(OH)F → O···Mg(OH)F + O_2_ (8)

O···Mg(OH)F + H_2_O → Mg(OH)F + 2 ·OH (9)

Path 2:

Mg(OH)F + O_3_ → Mg(OH···O_3_)F (10)

Mg(OH···O_3_)F + H_2_O → Mg(OH)F + ·OH + 1.5 O_2_ + H^+^ (11)

In conclusion, more acidic sites can not only improve the adsorption of pollutant molecules, but also promote the production of reactive oxygen species, and realize the degradation of organic pollutants at ambient temperature.

## 4. Optimization of Catalytic Activity

Catalytic oxidation can achieve effective degradation of VOCs, which is a promising method for VOC governance. Catalysts play a decisive role in the catalytic oxidation process. Researchers have been searching for efficient, stable, and selective catalysts through the optimization of catalyst configuration, element doping, acid/base modification, and other regulatory mediums. In the following, we summarize the improvement of the catalytic effect from aspects of support effect, coordination interaction, synergism, and process conditions, according to the classification of nanoparticle catalysts, cluster catalysts, and single-atom catalysts.

### 4.1. Nanoparticle Catalysts

#### 4.1.1. Nanoscale Metal Oxide Catalysts

**Figure 3:**
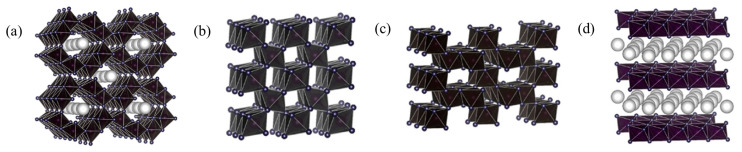
Crystalline structure schematic diagrams of (**a**) α-MnO_2_; (**b**) β-MnO_2_; (**c**) γ-MnO_2_; (**d**) δ-MnO_2_. Reprinted with permission: Elsevier 2020 [90].

In the catalytic oxidation of VOCs at ambient temperature, nanoparticle catalysts can be divided into nanoscale metal oxide catalysts and nano-metal supported catalysts. Transition metal oxides (Ni, Mn, Co, Ce) are the main nanoscale metal oxide catalysts, among which manganese oxide is the most widely studied. MnO_x_ catalysts have different structural forms of α-MnO_2_, β-MnO_2_, γ-MnO_2_, and δ-MnO_2_ (Figure 3). Moreover, the catalytic performance of MnOx is affected by various factors such as preparation method, crystallographic structure, morphology and tunnel structure [87].

**Figure 4:**
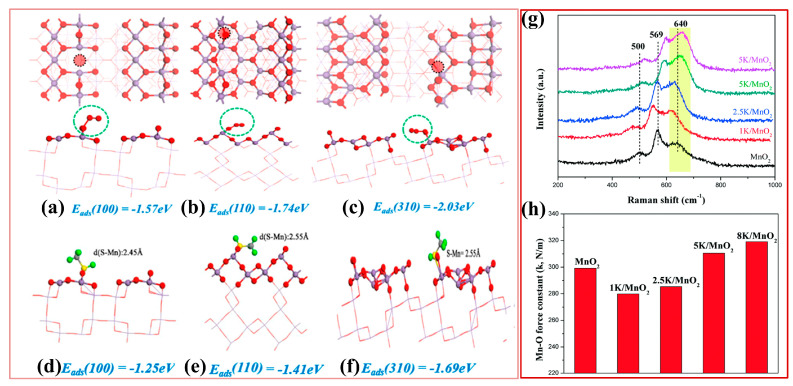
(**a**–**c**) Adsorption energy of O_3_ on oxygen vacancy of α-MnO_2_ with different exposed facets; (**d**–**f**) adsorption energy of S atom in CH_3_SH on oxygen vacancy of α-MnO_2_ with different exposed facets. Reprinted with permission: American Chemical Society 2020 [81]; (**g**) Raman spectra of the different MnO_2_ catalysts; (**h**) Mn–O bond force constant of various MnO_2_ catalysts. Reprinted with permission: Royal Society of Chemistry, 2018 [91].

Xu et al. [57] calcined MnO_x_ at different temperatures and obtained γ-MnO_2_, β-MnO_2_, and Bixbyte-like Mn_2_O_3_. Among the three catalysts mentioned above, mesoporous nanoparticle catalyst β-MnO_2_ had the highest surface oxygen species concentration and average oxidation state (AOS), showing long-term stable degradation effect of ethyl acetate at room temperature. Zhang et al. [59] prepared one-dimensional rod-like α-MnO_2_ and achieved complete degradation of formaldehyde at room temperature. The prepared α-MnO_2_ catalyst had a suitable tunnel structure, which was beneficial to formaldehyde adsorption. At the same time, α-MnO_2_ had abundant oxygen vacancies, which promoted the efficient conversion of adsorbed oxygen species into reactive oxygen species. Fundamentally, MnO_x_ catalysts with different crystal phases have specificity in crystal structure and tunnel structure, and have different oxidation activities for different pollutants. In addition, the catalytic activity of different crystalline phases of MnO_2_ also depends on the facets of MnO_2_. For example, He et al. [81] used crystal facet engineering to regulate the exposed facet of catalysts, and compared the catalytic oxidation activity of exposed (100) facet (100-MnO_2_), (110) facet (110-MnO_2_), and (310) facet (310-MnO_2_) nanowires for methyl mercaptan (Figure 4a–f). It was found that the 310-MnO_2_ facet exhibited the highest adsorption energy toward O_3_ and methyl mercaptan. Additionally, 310-MnO_2_ possessed a more intact surface for O_3_ than CH_3_SH capture, which first converted O_3_ into reactive oxygen species and then completely oxidized the neighboring adsorbed CH_3_SH. In metal oxides, metal or oxygen vacancies can improve the catalytic activity by adjusting the electronic structure of catalysts, improving the adsorption performance, reducing the reaction energy barrier, and forming unsaturated coordination sites. Furthermore, Rong et al. [91] doped base metal K into Birnessite-type MnO_2_ with abundant Mn vacancies and investigated the effect of base metal doping on the activity of catalysts. It was found that the doping of Mn:K = 1:1 maximized the structure and morphology of the catalyst. Due to the charge balance between K and Mn vacancies, the interaction between Mn vacancy and its surrounding “dangling” O atom was changed and weakened the Mn–O bond, which increased the lattice oxygen activity and lowered the formation energy of oxygen vacancies (Figure 4g,h). Similarly, Huang et al. [64] modified 3D-NiCo_2_O_4_ nanosheets using NaOH solution, effectively increasing the adsorption capacity and surface hydroxyl groups of the catalyst. Moreover, the rich surface hydroxyl groups optimized the reaction path of HCHO oxidation, which could directly react with intermediates to generate CO_2_ and H_2_O, achieving efficient catalysis of formaldehyde and high CO_2_ selectivity at room temperature.

#### 4.1.2. Supported Metal Nanoparticle Catalyst

- Support effect

**Figure 5:**
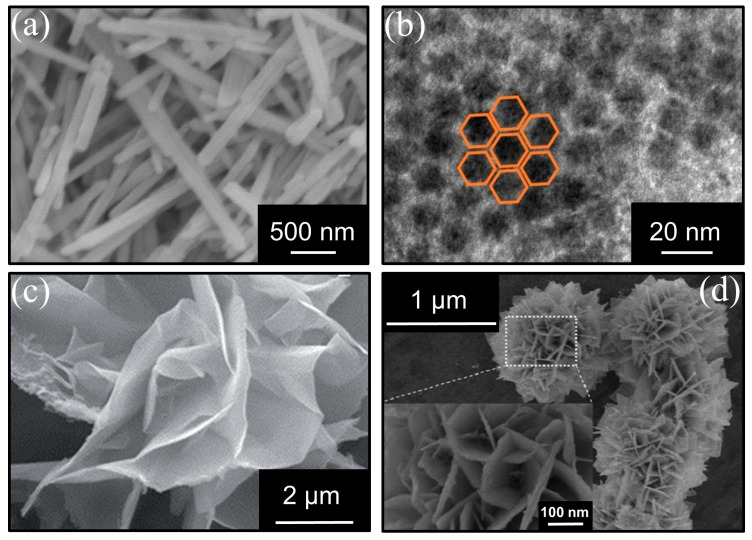
(**a**) SEM image of nanorod catalyst. Reprinted with permission: John Wiley and Sons, 2019 [59]; (**b**)TEM image of nanotube catalyst. Reprinted with permission: John Wiley and Sons, 2019 [72]; (**c**) SEM image of nanosheet catalyst. Reprinted with permission: Royal Society of Chemistry, 2016 [64]; (**d**) FESEM image of flower-like Pt/SnOx. Reprinted with permission: Elsevier, 2017 [68].

A supported metal catalyst is composed of a support and metal. As important components of catalysts, the pore structure and surface chemical properties of the support provide extensive positions for active site loading and pollutant adsorption [92]. Therefore, preparing the support with special configuration and optimizing the internal catalyst for effective mass transfer are important means to improve the catalytic activity. In the past few decades, researchers have fabricated multi-scale hierarchical nanostructured metal-supported catalysts such as nanorods [59], nanotubes [72], nanosheets [64], and so on (Figure 5a–c).

Considering that the multi-scale structure of support can effectively improve the diffusion of reactants and products in catalysts, Duan et al. [68] adopted the hydrothermal synthesis method to prepare flower-like Pt/SnOx composed of staggered petal-like nanosheets, with a specific surface area 7 times that of commercial SnOx, realizing the efficient dispersion of Pt nanoparticles and the efficient conversion of formaldehyde at room temperature (Figure 5d). Moreover, the interfacial electron transfer between supports and active components can be realized through structural regulation, which effectively changes the electronic properties of the catalyst surface and improves the electron transfer efficiency between ions [93]. Xu et al. [48] modified the activated carbon (AC) support with nitric acid, and found that appropriate nitric acid content could significantly increase the loading and dispersion of metal Mn on AC support, improve the content of acid oxygen-containing groups, and allow the supported metal Mn element to exist in the form of Mn_3_O_4_, which had the best lattice oxygen mobility and realized the catalysis of toluene at room temperature. To further improve the activity, selectivity, and stability of the catalyst in the reaction process, Zhu et al. [75] synthesized Pt/γ-Al_2_O_3_ catalyst using γ-Al_2_O_3_ with (110) facets exposed as support. The (110) facets led to fully exposed unsaturated tricoordinated Al (Al_3C_) sites on the Al_2_O_3_ surface, which in turn promoted the bonding between Pt and O atoms, resulting in a high dispersion of Pt particles on the catalyst surface. With the dispersion of highly active sites, oxygen molecules adsorption produced more active oxygen species, thereby realizing the complete oxidation of formaldehyde at room temperature. At the same time, Yang et al. [51] explored the influence of different SiO_2_/Al_2_O_4_ ratios in the Ag/ZSM-5 catalyst support on catalytic stability and found that the appropriate ratio would contribute to the slow adsorption and rapid desorption of ethylene pollutants on the support, and provide more acidic sites, so as to ensure the stability of the catalyst.

- Metal size effect

In fact, there is a strong metal–support interaction in supported catalysts. The configuration, composition and surface properties of supports interact with the size, morphology, dispersion, and stability of metal nanoparticles. Therefore, the structure and morphology of supported metals have significant effects on the catalytic activity.

First of all, for supported metal nanoparticle catalysts, the size effect of metal is often considered an important parameter affecting the structure and performance of catalysts. The electronic structure and geometric structure will change with the change in metal size. Yan et al. [67] prepared hierarchical Ni−Al hydrotalcite-supported Pt catalyst (Pt/NiAl-LDHs), which achieved high dispersion of Pt and exposed Pt (111) facets when metal Pt was loaded on the NiAl-LDHs support surface with nanoparticles approximately 3–4 nm in diameter. The high dispersion and surface morphology of Pt together led to the transfer of electrons from Ni to Pt, resulting in electron perturbation in Ni atoms to form defects, which could effectively promote the formation of more surface active oxygen and realize the complete oxidation of formaldehyde at ambient temperature.

- Metal loading

The metal loading of nanoparticles catalyst is also an important parameter affecting the catalytic activity. When the loading amount is too small, the metal active sites on the surface are few and the catalyst activity is weak. Instead, when the loading is too large, the metal active sites are prone to agglomeration, reducing the utilization of metal atoms, thereby reducing the activity of the catalyst. Aghbolaghy et al. [79] analyzed the effect of different Co loadings on the CoO_x_/γ-Al_2_O_3_ catalyst. Compared with the Co loading of 5% and 10%, the Co-2.5% catalyst showed excellent performance. The smaller loading made Co have better dispersion and smaller crystallite size, which increased the electron migration between cobalt atoms and supports, and altered the local environment. In the catalyst with the best loading, Co existed in the oxidation state of CoO, which had higher electron transfer ability and could better promote ozone decomposition and acetone removal.

- Polymetallic doping

**Figure 6:**
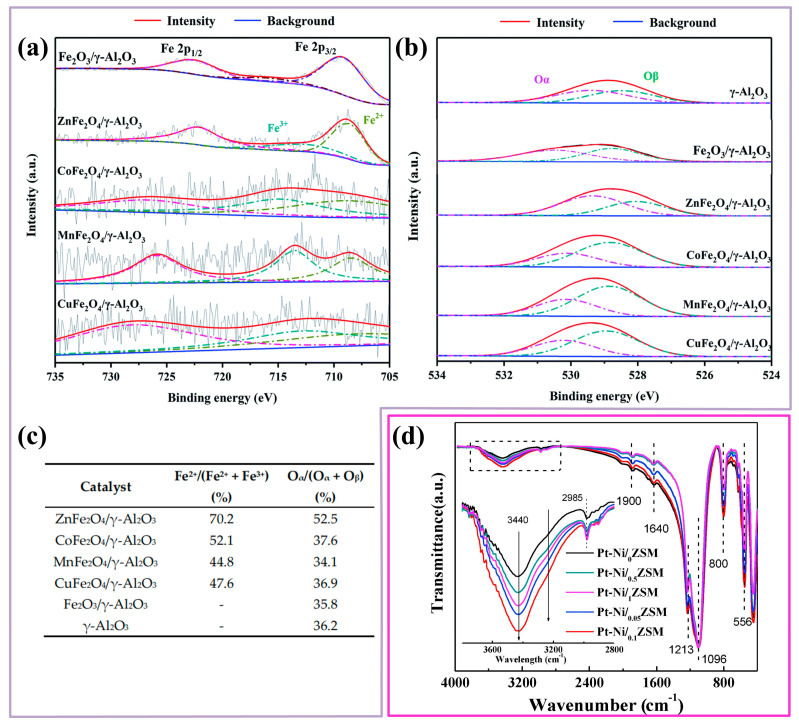
XPS spectra of (**a**) Fe 2p of Fe_2_O_3_/γ-Al_2_O_3_ catalyst and AFe_2_O_4_/γ-Al_2_O_3_ catalysts; (**b**) O 1s of γ-Al_2_O_3_, Fe_2_O_4_/γ-Al_2_O_3_ catalyst and AFe_2_O_4_/γ-Al_2_O_3_ catalysts; (**c**) XPS results of γ-Al_2_O_3_ and AFe_2_O_4_/γ-Al_2_O_3_ catalysts (O_α_: surface oxygen; O_β_: lattice oxygen). Reprinted with permission: Royal Society of Chemistry 2020 [44]; (**d**) FTIR spectra of Pt-Ni/_X_ZSM (peak at ca. 3440 cm^−1^ and ca. 1640 cm^−1^: the stretching and bending vibrations of OH; peak at ca. 2985 cm^−1^: terminal OH). Reprinted with permission: Elsevier 2018 [74].

Noble metals (Pd, Pt, Au, Ru, and Ag), transition metals (Co, Mn, Ce, Al, La, Zn, Ni, and Cu), and their oxides are mainly used as active sites of supported metal catalysts. In order to improve the performance of catalysts, many studies have attempted to prepare polymetallic composite catalysts or doped the catalysts with metals or metal oxides, utilizing the cooperation of multiple metals to achieve the excellent activity of catalysts. Jiang et al. [44] prepared a variety of binary transition metal catalysts using nano ferrites (AFe_2_O_4_, A=Zn, Co, Mn, Cu), among which ZnFe_2_O_4_/γ-Al_2_O_3_ could effectively regulate the valence state of Fe on the catalyst surface. The transformation of Fe^3+^ to Fe^2+^ could introduce lattice oxygen vacancies (Ovs) on the catalyst surface, and the higher the proportion of O_α_ in the oxygen element distribution, the stronger the adsorption performance of the catalyst on the O_3_ to produce more active oxygen species (Figure 6a–c). At the same time, the combination of Zn and ferrite made the catalyst show the strongest Lewis acid sites and Brønsted acid sites, which enhanced the adsorption capacity of toluene, so as to achieve stable and efficient degradation at room temperature. In addition, Ding et al. [74] added appropriate nickel cations to the Pt/ZSM-5 catalyst. Without changing the physical structure of the catalyst, through ion exchange and the restriction effect of Ni ions, it not only reduced the loss of Pt, but also effectively increased the density of hydroxyl groups around Pt active sites. Finally, the oxidation property of formaldehyde is enhanced (Figure 6d). Fan et al. [94] prepared a highly dispersed FeO_x_-CeO_x_ nanocatalyst supported on SBA-15 molecular sieve. During the process of doping Fe on SBA-15 loaded with CeO_2_, the Fe-Ce-O solid solution was formed on the catalyst surface due to the surface defects of CeO_2_ and the different concentrations of Fe inside and outside CeO_2_. The migration of Fe^3+^ on CeO_2_ replaced part of Ce^4+^ and increased the ratio of Ce^3+^, which was more conducive to the generation of oxygen vacancies and the mobility of oxygen. Alkali metal (Na, K, etc.) doping, as a common type of doped metal, has great impact on the structure, morphology, and activity of catalytic materials. Song et al. [69] constructed Pt/NaY nanocatalyst modified by K. The addition of K could regulate the electronic structure of catalyst surfaces and enhance the hydroxyl density of catalyst surfaces, the adsorption capacity of pollutants, and the low-temperature reduction performance. The complete oxidation of formaldehyde at ambient temperature was achieved by the modified catalyst. To summarize, it can be found that the size effect of metal atoms, structural regulation of polymetallic doping, and the oxidation state of metal oxides at the active site are commonly used modification methods to improve the catalytic activity.

### 4.2. Metal Cluster Catalysts

A cluster refers to a class of compounds containing two or more metal atoms and metal–metal bonds or other metal coordination bonds [95]. A metal cluster catalyst is an ideal catalytic oxidation material at room temperature because of its rich active sites and strong structural designability. Cluster catalysts also have size, surface, and structure effects. Uniquely, a metal cluster catalyst can determine the constituent atoms and structures of metal active sites, and has discrete electronic energy levels, ultra-high specific surface area, chemical activity, etc. [96,97]. Researchers have designed a series of novel stable and efficient cluster catalysts by optimizing the structure and function of catalysts. Tian et al. [53] prepared Pt/FeO_x_ catalyst that supported platinum clusters. Through model construction, it was determined that the three-atom Pt_3_/Fe_2_O_3_ catalyst model was the most stable. Furthermore, the Pt_3_ clusters were highly dispersed on the catalyst support, and the mole ratio of Pt^0^/Pt^2+^ in the metal clusters was 1, providing the best activity for the catalysts. For cluster catalysts, different configurations such as two-dimensional and three-dimensional can be presented according to the size and number of metal atoms. At the same time, there is a direct interaction between some atoms in clusters and catalytic supports, and clusters will change with the configuration of supports [98].

### 4.3. Single-Atom Catalysts

Single-atom catalysts (SACs), as a research hotspot in recent years, have fully exposed active sites and ultra-high atomic utilization compared to traditional catalysts. The lower coordination configuration and unsaturated state help SACs decrease the intrinsic activating energy of reactants. Zhang et al. [99] used the one-pot hydrothermal method to load single-atom Pt onto MnO_2_, which improved the catalytic activity of toluene at room temperature. Mn^3+^ and oxygen vacancies in catalyst support MnO_2_ had a highly effective stabilizing effect on single-atom platinum, greatly activating surface oxygen species and promoting the generation of hydroxyl radicals. In SACs, single atoms and supports form strong interactions through electron transfer or interface bonding, so modifying the supports and adjusting their electronic structure can promote catalytic activity and stability [100,101]. Cui et al. [54] carried out Na doping treatment on Pt/TiO_2_ SAC, which changed the neighbor coordination among O, Ti, and Pt. The strong interaction between Pt and Na atoms resulted in electron transfer and reduced the activation barrier on Na-Pt/TiO_2_, which generally improved the stability of the system. Actually, as metal particles decrease to the size of single atoms, the high surface energy and mobility of the metal surface will cause agglomeration and formation of nanoclusters in the preparation process of the catalyst [102]. Therefore, it is important to select appropriate catalyst supports and form spatial confinement by atomic doping in single-atom catalysts. Zhang et al. [4] tried to atomically disperse Y or La on Birnessite-type MnO_2_. With the doping of alkaline earth metals, the specific surface area of MnO_2_ increased and more Mn defects were produced in the structure, around which the lattice oxygen was more easily activated and converted into the surface active oxygen specie. In addition, the interaction between incorporated Y or La atoms and support could improve the moisture resistance and structural stability of the catalyst.

### 4.4. Regulation of Reaction Conditions

The catalytic oxidation reaction at ambient temperature is an extremely complex process affected by many factors. To give full play to the activation of catalysts and achieve the maximum oxidation of pollutants, different reaction conditions need to be formulated for different catalysts. Here, we have summarized the optimal initial pollutant concentration, reaction space velocity, relative humidity, and oxidation atmosphere of different catalysts for the degradation of different pollutants. The space velocity and the initial concentration of pollutants are limited by the activation performance of catalysts. It is generally believed that the higher space velocity and initial concentration, the higher catalyst activity and the better treatment capacity. In addition, optimal exhaust gas humidity and other gas assistance can effectively improve the oxidation activity of catalysts.

#### 4.4.1. Initial Concentration

**Figure 7:**
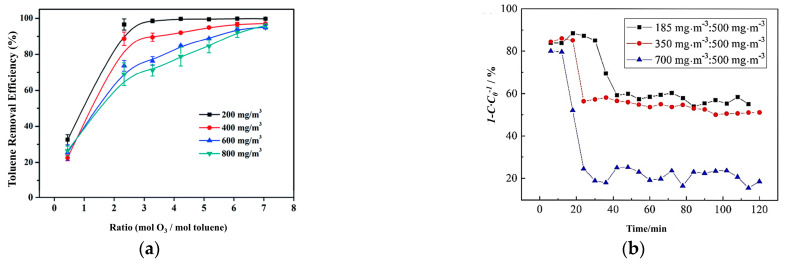
(**a**) Removal of toluene at different initial toluene concentrations with ZnFe_2_O_4_/g-Al_2_O_3_ catalyst. Reprinted with permission: Royal Society of Chemistry 2020 [44]; (**b**) the effects of inlet concentrations of ethyl acetate on catalytic performance of Pd/ACF catalyst (ozone concentration: 500 mg·m^3^). Reprinted with permission: Elsevier 2018 [56].

In practical engineering applications, the concentration of VOCs from different emission sources varies greatly. Selecting the appropriate initial concentration is a necessary step to evaluate the catalytic activity. It is generally believed that the catalyst’s active sites limit the maximum initial concentration of VOCs. In the heterogeneous catalytic reaction, if the initial concentration is too low, the adsorption rate is lower than the reaction rate, and chemical adsorption is the main determinant. With too high an initial concentration, the adsorption rate is higher than the reaction rate, and the surface reaction and desorption rate are the decisive determinants. When the adsorption rate is close to the reaction rate, the initial concentration of VOCs is the most appropriate, and the pollutant degradation efficiency is the highest [103]. Furthermore, the significant difference between VOC removal rate and CO_2_ selectivity often occurs during the degradation of low-concentration pollutants at ambient temperature, which means that VOCs are not completely mineralized and/or partly transformed into certain intermediates [4]. Jiang et al. [44] selected the toluene simulated waste gas with initial concentrations of 200, 400, 600, and 800 mg·m^3^ for experiments based on the concentration of toluene in actual industrial waste gas (Figure 7a). Under equal conditions, the higher the concentration of toluene, the worse the degradation effect, and the degradation rate of toluene showed a gradual positive correlation with the O_3_ mol dosing ratio. This is mainly due to the insufficient supply of reactive oxygen species generated by the decomposition of ozone on the catalyst surface, and as it is difficult for the excess toluene to undergo an oxidation reaction, more O_3_ was required to maintain the adsorption equilibrium on the catalysts. In addition, Cui et al. [56] found that the smaller the initial concentration, the slower the adsorption saturation of ethyl acetate on ACF, the more ozone was exposed per unit time, and the more ethyl acetate was degraded, under the same ozone concentration (Figure 7b). There is adsorption competition between ozone and ethyl acetate on the surface of catalysts. The more active oxygen species produced by ozone adsorption and decomposition, the better degradation effect of pollutants.

#### 4.4.2. Space Velocity

**Figure 8:**
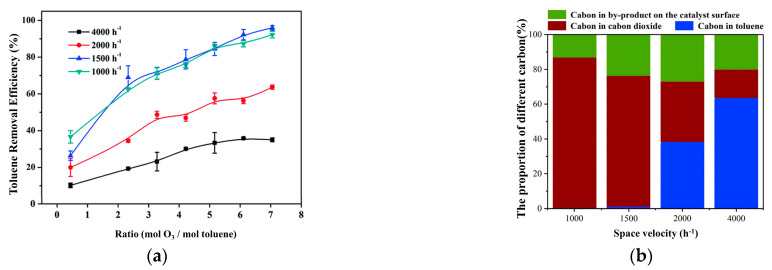
(**a**) Removal of toluene at different space velocities with ZnFe_2_O_4_/g-Al_2_O_3_ catalyst; (**b**) impact of space velocity on carbon balance. Reprinted with permission: Royal Society Chemistry 2020 [44].

In order to describe the gas flow through the catalyst per unit time, we usually use gas hourly space velocity (GHSV) and weight hourly space velocity (WHSV) in ambient temperature catalytic oxidation applications. The increase in space velocity means that the amount of gas that goes through the catalyst is large. This implies that if the space velocity is smaller, the contact time will become much longer, which increases the reaction conversion rate. Therefore, it can be seen that reducing the space velocity is beneficial to improve the catalytic effect, but the lower space velocity requires more catalysts under the same amount of pollutants, which weakens the economy of the treatment process. Xu et al. [57] studied the effect of GHSV on the catalytic ozonation of β-MnO_2_. Lower GHSV led to higher CO_2_ selectivity, while under higher GHSV, more intermediate products formed in the catalytic ozonation of ethyl acetate. Similarly, Jiang et al. [44] summarized the effect of the space velocity on the catalytic reaction. As the space velocity decreased, the toluene degradation rate increased (Figure 8a). However, when the space velocity was large, the adsorption of toluene by the catalyst was limited, which limited the degradation ability of the catalytic ozonation process for toluene. A large amount of by-products adhere to the catalyst surface, which affects the degradation rate of toluene (Figure 8b). These results all indicate that lower space velocity can promote the complete oxidation reaction and reduce the accumulation of intermediate products on the catalyst surface.

#### 4.4.3. Oxidation Atmosphere

**Figure 9:**
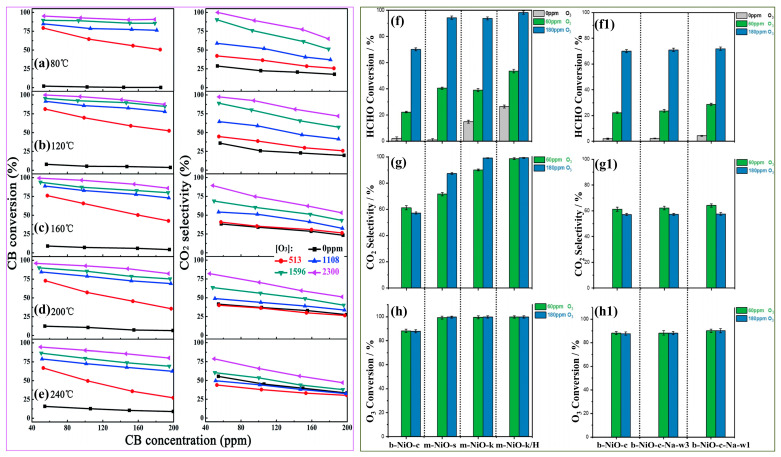
(**a**–**e**) Temperature dependence of chlorobenzene catalytic oxidation over MnO*x*/CNTs with and without O_3_. Reprinted with permission: Royal Society of Chemistry, 2015 [80]; (**f**,**f1**) HCHO conversion, (**g**,**g1**) CO_2_ selectivity, and (**h**,**h1**) O_3_ conversion over different catalysts at room temperature. Reprinted with permission: Elsevier 2018 [71].

As a highly oxidizing gaseous oxidant, ozone is often used as an assistant gas to reduce the temperature for degrading volatile organic compounds [82]. At present, there has been much research on ozone catalytic oxidation of VOCs, which has proved that it has the advantages of high pollutant degradation efficiency, low catalytic reaction temperature, environmental friendliness, and is considered to be a promising pollutant treatment technology [104]. In fact, catalytic ozonation behavior is also related to the types of pollutants and the properties of catalysts. Liu et al. [11] found that the oxidation activity of VOCs assisted by ozone depended on the strength of carbon-containing bonds, ranked as alcohols < aldehydes < aromatics < ketones < acetates < alkanes. Moreover, MnO_2_-based catalysts were considered to be the best catalyst for catalytic ozonation. In addition to the characteristics of pollutants and catalysts, the mixing ratio of pollutants and ozone, and the reaction temperature also affect the catalytic activity in the catalytic ozonation reaction. Machniewski et al. [105] found that the extent of toluene mineralization increased with temperature up to 60 °C, when the ozone/toluene molar ratios were less than 20. If the reaction temperature continued to rise, it was necessary to increase the ozone/toluene molar ratio to achieve the maximum mineralization of toluene due to the “unproductive” loss of ozone. Similarly, Jin et al. [80] found that under the synergistic effect of MnO_x_/CNT catalyst and ozone, the higher the ozone concentration, the longer the interaction time between catalyst and ozone. At the same time, more active oxygen species were provided on the surface of catalyst, which made it easier to realize the oxidation of chlorobenzene at low temperature (Figure 9a–e). In fact, increased catalytic activity with the assistance of ozone is achieved through the decomposition of ozone to supply more active oxygen species. Therefore, oxygen can also be used as an assistant gas to enhance catalytic activity. Wang et al. [71] compared the different catalytic effects of ordered mesoporous nickel oxide catalysts on formaldehyde under assistant gases of oxygen and ozone (Figure 9f–h,f1–h1). Although formaldehyde has been mineralized, well assisted by both gases, ozone could achieve a higher formaldehyde conversion rate and CO_2_ selectivity at ambient temperature. The reason was that when oxygen was used as the oxidant, the catalyst surface etched by alkaline solution was conducive to the regeneration of hydroxyl groups and the oxidation of formaldehyde, while the sodium surface modification was ineffective for HCHO catalytic oxidation with ozone. When ozone was used as the oxidant, NiO defects on the catalyst surface and extra adsorbed active oxygen species, which had higher activity, contributed almost exclusively to improving the reaction activity and CO_2_ selectivity.

#### 4.4.4. Humidity

**Figure 10:**
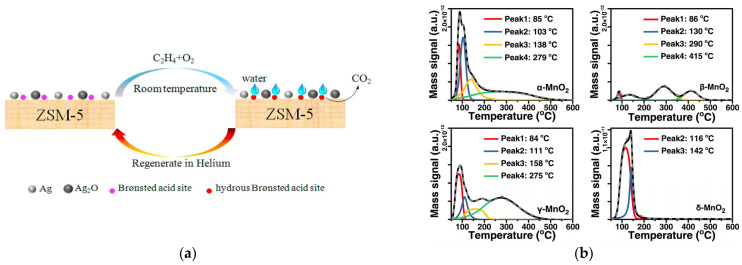
(**a**) The effect of water on the decreased catalytic activity of Ag/ZSM-5 for ethylene oxidation at room temperature. Reprinted with permission: Elsevier 2018 [51]; (**b**) the H_2_O-TPD profiles of α-MnO_2,_ β-MnO_2,_ γ-MnO_2_, and δ-MnO_2_. Reprinted with permission: Elsevier 2022 [107].

Water vapor widely exists in the exhaust gas of various industries. Considering the practical engineering applications, it is important to discuss the influence of humidity on catalytic activity. In previous studies, some researchers posited that H_2_O would be adsorbed on active sites of porous catalysts, inhibiting the adsorption and the further catalytic oxidation of VOCs [106]. Yang et al. [51] compared Ag/ZSM-5 adsorbed H_2_O with fresh Ag/ZSM-5, finding that H_2_O had strong adsorption on Brønsted acid sites and competed with ethylene on the same adsorption sites, which led to catalyst poisoning and deactivation. However, combined with the adsorption and desorption kinetics of H_2_O, it was found that H_2_O has relatively slow adsorption and rapid desorption characteristics at Brønsted acid sites of Ag/ZSM-5, which helped to maintain the stable activity of catalyst at room temperature (Figure 10a). Therefore, the role of water in catalytic oxidation cannot be generalized. When H_2_O molecules participate in the catalytic process of VOCs, the catalytic performance is improved to a certain extent. For example, water vapor can replenish the consumed surface hydroxyl and accelerate the desorption of by-products on catalyst surfaces, etc. [63,65]. Zhao et al. [107] also found that positive or negative effects of water vapor on catalytic activity were related to the reaction temperature and catalyst structure. The results showed that the Mn-O-Mn stretching bond in the MnO_2_ lattice with the special structure was conducive to the generation of a large amount of associative adsorbed H_2_O. At a moderate temperature of 70~100 °C, the associated adsorbed H_2_O firstly adsorbed on the surface of MnO_2_ and bonded with acetaldehyde to form a hydrogen bond, and then completely oxidized to CO_2_ under the action of active hydroxyl. However, when the catalytic reaction temperature was too high (>100 °C), the hydrogen bond between H_2_O and acetaldehyde disappeared, but the competitive adsorption relationship was formed. At this point, H_2_O played a negative role in the degradation of pollutants (Figure 10b). To summarize, it can be found that the effect of water molecules on VOC catalytic oxidation has a complex mechanism, which needs specific analysis for different materials, systems, and pollutant molecules.

## 5. Conclusions and Outlook

As an energy-saving and environmentally friendly VOC purification technology, ambient temperature catalytic oxidation has significant development space, whether in terms of the development of efficient catalysts or the regulation of oxidation process conditions. This article reviews recent studies on the catalytic oxidation of VOCs at ambient temperature and draws the following conclusions:

- Nanoparticle catalysts are widely used in ambient temperature catalytic oxidation, which often uses a variety of silicon-based materials, activated carbon, and metal oxides as catalyst supports, and noble metals, transition metals, and transition metal oxides as active components.
- The oxidation activity of the ambient temperature catalytic reaction mainly comes from the reactive oxygen species (·O_2_^−^,·OH, ^1^O_2_), which are mainly formed by the activation of ozone, oxygen, and water in metal active sites, defects, and acid/base sites.
- Configuration optimization, metal doping, defect engineering, acid/base modification, and other control methods are common strategies for preparing catalysts. Configuration optimization can provide a larger specific surface area, more active facets, more unsaturated coordination sites, and higher electron migration ability. In addition, metal doping, defect engineering, and acid/base modification can effectively adjust the coordination environment of catalysts and affect the metal charge of the active centers, thereby improving the dispersion, stability, and catalytic activity of the active sites.
- The catalytic oxidation reaction at ambient temperature is an extremely complex process. The initial concentration of pollutants, reaction space velocity, oxidation atmosphere, and relative humidity are important factors affecting the catalytic reaction efficiency. The space velocity and the initial concentration of pollutants in the reaction conditions are mainly limited by the activation performance of catalysts. In contrast, the oxidation atmosphere and humidity affect the activity of catalysts during the reaction of ambient temperature catalytic oxidation.

In addition, we also found the limitations of ambient temperature catalytic oxidation technology. The activity of general nanoparticle catalysts is insufficient. Hereby, the development of normal temperature catalytic mainly focuses on the removal of formaldehyde pollutants, while there are few studies on pollutants with benzene ring, C=C, C-Cl, C-S, and so on. Moreover, although the ambient temperature catalytic reactions reviewed above do not need the assistance of additional energy such as high temperature, high voltage, discharge, and ultraviolet light, most catalytic reactions need enough ozone molecules and oxygen molecules to supplement the reactive oxygen species in order to improve the catalytic reaction activity. These problems are mainly due to the lack of activity and stability of catalysts, which makes it difficult to achieve sustainable and efficient catalytic effects.

To solve such problems and realize the complete oxidation of volatile organic compounds at ambient temperature and air atmosphere, we should focus on increasing reactive active sites, as well as improving the high efficiency, stability, and universality of catalysts. Taking single-atom catalysts (SACs) as the starting point for realizing catalysis at ambient temperature, first, adjust the coordination configuration and atomic unsaturated state of catalysts. Second, focus on the unique role of quantum size effect on electron transfer within the catalyst. Third, pay attention to the strong metal–support interaction formed by electron transfer or interface bonding between supports and metals. As derivative catalysts of SACs, diatomic catalysts (DACs) and single cluster catalysts (SCCs) can adjust the electronic structure of catalytic active sites by using the synergistic interaction between adjacent metal sites, which is more efficient than SACs. Fully exposed metal cluster catalysts (FECCs) also have unique advantages. All the atoms are bulk-phase atoms without coordination number saturation, which can provide abundant surface active sites for catalytic reactions and achieve better catalytic performance than ordinary SACs. The development and application of these new catalysts will contribute to the realization of catalytic oxidation at ambient temperature without any assistance conditions.

The stability and durability of the catalysts still need to be concerned. The active substances on catalyst surfaces often agglomerate, migrate, or even fall off, leading to the deactivation of catalysts. Furthermore, water vapor and intermediate products will have competitive adsorption with pollutant molecules, resulting in the loss of active sites and the degradation of catalytic performance. Under the carbon peaking and carbon neutrality goals of green production and efficient utilization, we still need to invest more efforts to prepare stable, efficient, and selective catalysts, and explore the general mechanism of catalytic oxidation at ambient temperature. It is our common purpose to realize the green governance of volatile organic compounds.

## Data Availability

Not applicable.
